# Oral Transfer of Anti-Aging Substances: Key Chemical Found in Reproductive Caste of Termites

**DOI:** 10.3390/ijms26041543

**Published:** 2025-02-12

**Authors:** Xin Peng, Zahid Khan, Yanan Dong, Lian-Xi Xing

**Affiliations:** 1 Key Laboratory of Resource Biology and Biotechnology, Xi’an International University, Xi’an 710077, China; 15209247629@163.com; 2 Key Laboratory of Resource Biology and Biotechnology in Western China, College of Life Sciences, Northwest University, Xi’an 710069, China; 3 School of Ecology and Environment, Northwestern Polytechnical University, Xi’an 710072, China; dyn0489@mail.nwpu.edu.cn

**Keywords:** *Reticulitermes labralis*, longevity, IIS pathway and RAS pathway, trophallaxis compounds

## Abstract

With the rapid increase in global population aging, the incidence and mortality rates of age-related diseases are rising, becoming a worldwide issue. Therefore, researching and discovering natural compounds with anti-aging properties is crucial. Social insects such as termites exhibit significant differences in lifespan between reproductive and non-reproductive castes. Reproductive castes are exclusively fed by worker termites through trophallaxis, providing a convenient model for the discovery of natural anti-aging compounds. This thesis systematically investigates the trophallactic fluid among different caste members of termite *Reticulitermes labralis*. A total of 1028 metabolites were identified in the trophallactic fluid, seven of which have been validated in the KEGG database to possess anti-aging functions. This indicates that the trophallactic fluid of termites indeed contains natural compounds that promote longevity. Using the “fishing method”, we successfully screened out potential life-extending compounds, including IDA (trans-3-indoleacrylic acid). Preliminary experimental results showed that IDA influences lifespan by modulating the IIS (insulin/insulin-like growth factor signaling) pathway and the RAS pathway. Notably, the modulation of the IIS pathway by IDA does not require the involvement of foxoa. Our research findings suggest that the extended lifespan of reproductive termites is diet-related and that the lifespan-extending effects of these nutritionally regulated natural compounds are conserved across different taxa.

## 1. Introduction

Over the millennia of human development, life expectancy has increased dramatically as a result of modern science (diet, lifestyle, and healthcare improvements), e.g., Europe and developed regions such as Japan have extended from 25 years in the Bronze and Iron Ages to over 80 years. However, increased longevity is often accompanied by physical aging, making the chances of developing age-related diseases such as cardiovascular disease, neurodegenerative diseases, and cancer much higher and the quality of life much lower [1]. According to a study by the American Heart Association, by 2030, more than a fifth of the world’s population will be elderly [2]. Therefore, it is critical and urgent to find effective, side effect-free, easy-to-use medicines and other means to enable human beings to live more healthily in the old age.

For more than a century, a variety of classical animal models have been used to explore the regulatory pathways behind aging in the search for anti-aging pathways without side effects. Most of the pathways associated with longevity have been largely identified [3,4,5]. For example, the insulin/insulin-like growth factor (IGF) signaling (IIS) pathway and the serine/threonine kinase mechanistic target of rapamycin complex (mTORC) are crucial in regulating cellular metabolism and growth. mTOR, by linking nutrient availability and metabolic pathways to longevity, simultaneously contributes to aging through the inhibition of autophagy [6,7,8,9]. AMPK, the nutritional pathway that antagonizes mTOR, has also been demonstrated to extend lifespan to some degree upon activation [10,11]. One study also found that insulin signaling is influenced by the renin–angiotensin system (RAS), which was observed to be upregulated along with insulin signaling in the brains of aged rats [12]. These effects can be counteracted by AT2 [13], prolonging healthy life [14]. Interestingly, recent studies have also suggested that the β2-adrenergic system may be involved in human longevity and diseases such as heart failure [15,16,17].

As research has evolved, it has been revealed that the lifespan of metazoans is influenced not only by genetic factors but also by environmental factors, including temperature, food intake, and nutrition [18,19]. Dietary interventions such as anti-inflammatory nutrients, calorie restriction, and the addition of angiotensin II receptor blockers have also been clinically shown to play an important role in regulating the aging process and longevity [20,21,22,23]. Both intermittent fasting and caloric restriction can improve metabolic and inflammatory parameters in humans, and both are promising avenues for attenuating aging, nutritional processes, and neurodegenerative diseases [24,25,26]. But calorie restriction and intermittent fasting interventions mean a long-term 35–45% reduction in calorie intake from the usual diet, which is almost impossible to adhere to in most people’s daily lives [27,28]. Altogether, dietary changes can indeed affect the length and quality of life, but there is a need to find a way to extend a healthy life without so many limitations.

Research on natural anti-aging in model organisms to find natural compounds with anti-aging effects in their diets may yield breakthroughs. Although traditional model organisms facilitate experiments due to their extreme generational characteristics, their excessively short lives render them unsuitable as samples for this study [29,30]. Social insects share a common set of genetic templates throughout the nesting colony, but reproductive and worker classes differ dramatically in longevity. Thus, social insects are a natural new biological model for studying aging [31]. Recent studies on black garden ant, *Lasius niger* and termite *R. speratus* have shown that the dietary composition of the members of a nest colony with different behavior is biased, and that the workers also have discriminative trophallaxis, with the king and queen receiving different food compositions during the feeding process [31,32]. Due to the unique characteristics of the termite reproductive caste, exploration thereof is expected to reveal natural compounds and regulatory mechanisms associated with aging and longevity.

Recent studies on the life history of the reproductive caste have also found that changes in termite behavior can also affect changes in brain size, with the reproductive caste having a significantly larger brain than the other castes, which is different from other animals, and we speculate that this change may be related to dietary patterns [33]. Given the specificity of termite life histories, we hypothesize that studies of the composition of the trophallaxis fluid of various castes of termites will hopefully elucidate the relationship between diet and longevity. In order to examine this issue, the species of compounds in the feeding solution of each grade in termite colonies were explored using non-targeted liquid chromatography-tandem mass spectrometry (LC–MS/MS). We also screened compounds with anti-aging properties from termite longevity-caste diets by targeting specific AT2 receptor probes associated with longevity and brain growth.

## 2. Result

**Figure 1:**
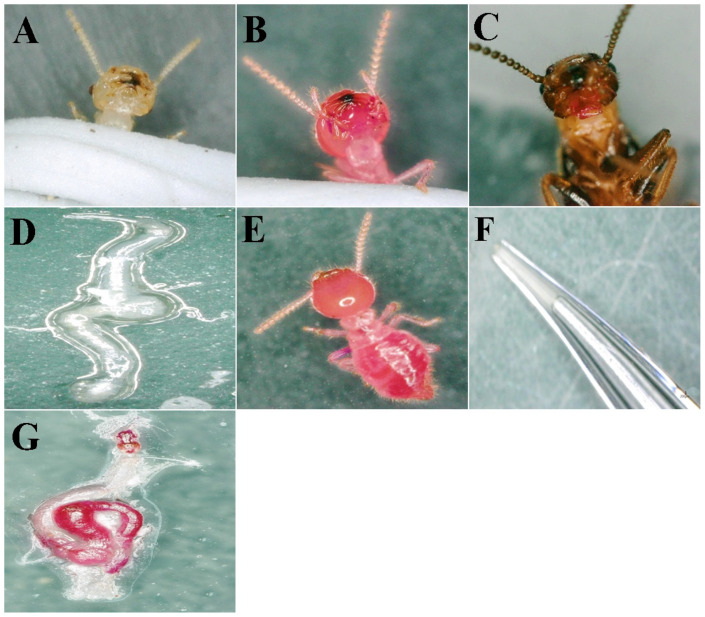
Staining position and sampling from termite worker. (**A**) Worker oral fluid. (**B**) Third instar worker-stained oral fluid. (**C**) Primitive queen oral fluid. (**D**) Termite gut. (**E**) Third instar worker that has eaten stained food. (**F**) Schematic diagram of test tube sucking up oral fluid. (**G**) Intestines of termites that have eaten colored foodstuffs.

To determine and confirm the location of the trophallaxis fluid, we stained the termite food. We conducted an experiment in a primary colony where the larvae were fed only by the king and queen. Figure 1A demonstrates that after immobilizing worker termites, blowing or abdominal squeezing—methods that mimic natural stimuli in the termites’ lifestyle—can successfully induce them to regurgitate the trophallaxis fluid. Figure 1D illustrates the gut structure of the round-lipped subterranean termite, providing morphological insights into the formation and transmission pathways of regurgitated food (i.e., partially digested food exchanged between termites). By examining Figure 1B,C,E–G, we can observe liquid samples obtained from the termite body through methods such as squeezing after staining treatment. These samples primarily originated from the crop of the termite. In order to ensure the validity and representativeness of the experimental data, two methods were used to extract the termite food: one was to directly aspirate the liquid from the mouth, and the other was to extract the liquid stored in the crop through dissection. The combination of these two methods provided sufficient biological samples for subsequent metabolomic analysis.

### 2.1. Metabolomic Analysis Results

**Figure 2:**
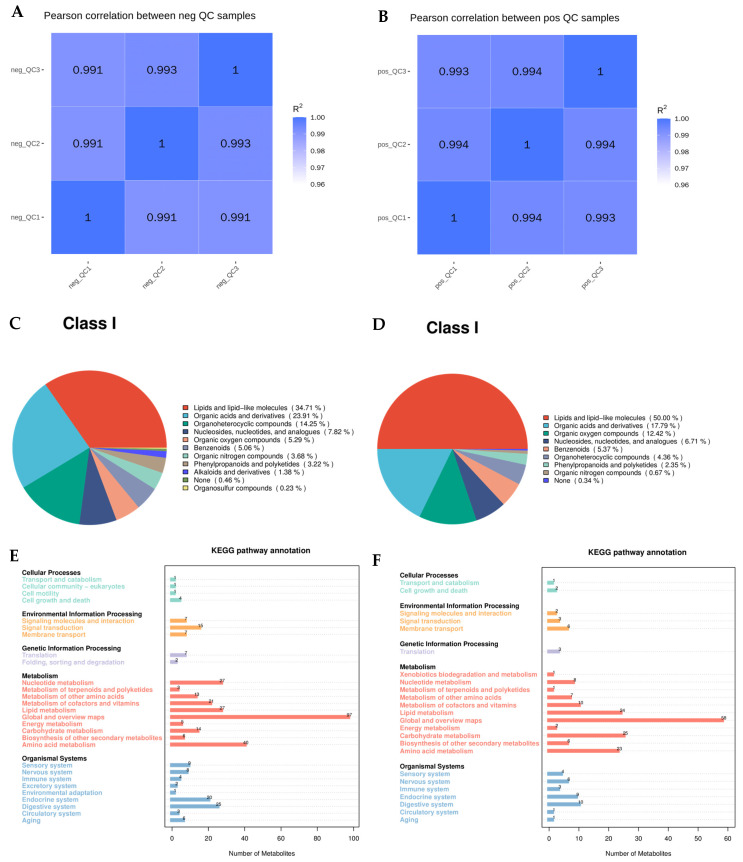
Metabolomic results. (**A**) Positive ion mode metabolite QC values. (**B**) Negative ion mode metabolite QC values. (**C**) Positive ion mode metabolite pie chart. (**D**) Negative ion mode metabolite pie chart. (**E**) Positive ion mode metabolite KEGG annotation map. (**F**) Negative ion mode metabolite KEGG annotation map.

Based on the relative quantification values of metabolites, we calculated the Pearson correlation coefficients between quality control (QC) samples. The Pearson correlation coefficient is a statistical measure that assesses the strength and direction of the linear relationship between variables, ranging from −1 to 1. A value closer to 1 indicates a stronger positive correlation, meaning higher similarity between samples. The correlation analysis results of the QC samples in this experiment confirmed that the experimental data were accurate and reliable (Figure 2A,B). Figure 2C,D present the statistical results of the comprehensive metabolite analysis detected by mass spectrometry in both positive and negative ion modes. Figure 2E,F show the classification and functional annotation of metabolites from the total sample using the KEGG database. Among these, seven compounds were directly associated with longevity.

### 2.2. Functional Substances Identified from Differential Metabolites

**Figure 3:**
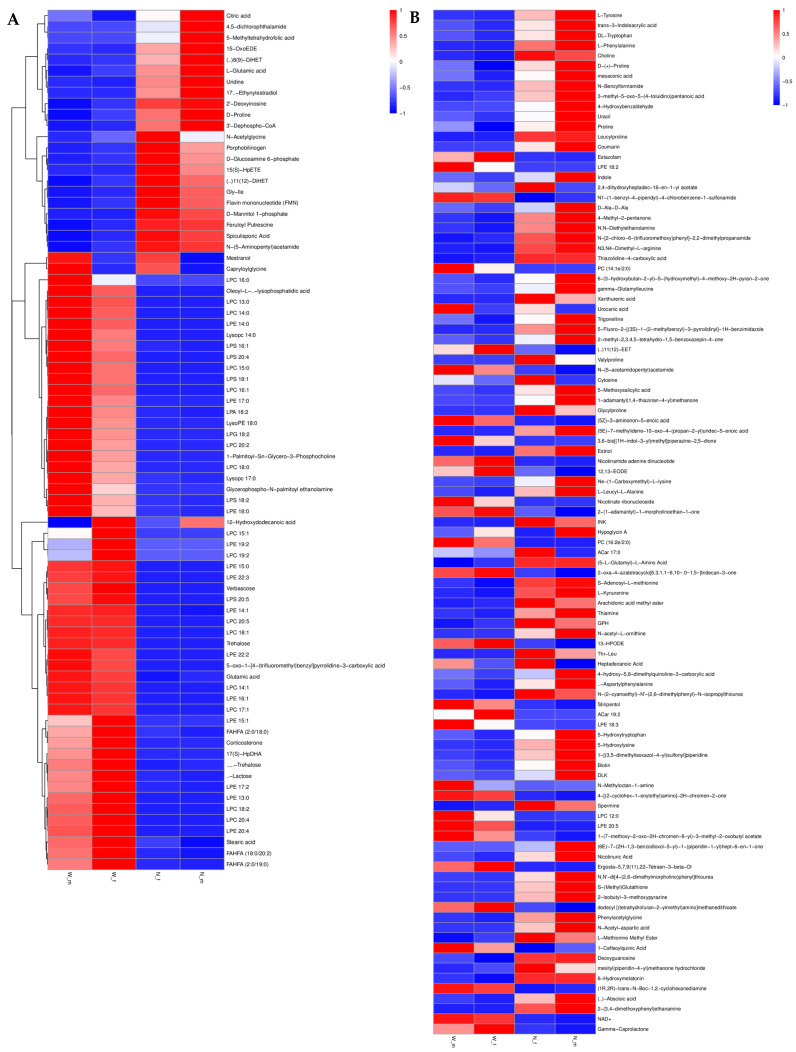
Different metabolites of different castes of termite *R. labralis.* (**A**) Difference in negative ion pattern metabolites of different castes of *R. labralis*. (**B**) Difference in positive ion pattern metabolites of different castes of *R. labralis*. The redder the color, the higher the content. See the Supplementary Materials for details.

We conducted a comprehensive identification and analysis of multiple substances in the trophallactic fluid of termites that may have positive lifespan regulatory effects. These substances were broadly classified into three categories based on their composition and function: carbohydrates, hormones, and known metabolites with lifespan-extending properties. Carbohydrates detected in the negative ion mode included α-lactose, trehalose, D-ribose, D-fructose, D-glucose, fructose-1,6-bisphosphate, D-threose, stachyose, and sucrose. Carbohydrates identified in the positive ion mode included tagatose, maltotetraose, and maltotriose. Tagatose, a rare monosaccharide, is of particular interest due to its low-calorie value, blood glucose-stabilizing effects, and antioxidant properties. The levels of these carbohydrate substances were found to be upregulated in reproductive caste termites compared to worker termites, which aligns with previous studies [34]. Hormones identified in the negative ion mode included 13,14-dihydroprostaglandin E1, prostaglandin A3, estrone, estrogen, prostaglandin A1, and 8-iso-15-keto-prostaglandin E2. Hormones detected in the positive ion mode included methyltestosterone, testosterone, 17α-hydroxyprogesterone, and bicyclo-prostaglandin E2. The trends in hormone levels varied between reproductive and worker castes. Some hormones showed decreased expression in the reproductive caste compared to the worker caste. Functional compounds identified in the negative ion mode included methadone-D9, pantothenic acid, docosahexaenoic acid (DHA), 10-hydroxydecanoic acid, royal jelly acid, and (2E)-4-hydroxy-3,7-dimethyl-2,6-octadien-1-yl β-D-glucopyranoside, etc. Functional compounds detected in the positive ion mode included L-carnitine, nicotinamide, trigonelline, 2-hydroxycinnamic acid, 4-(4-morpholinyl)benzoic acid, methyl palmitate, aloesin, 9-oxo-ODE, eucalyptol, 3,5-dihydroxyphenylglycine, C_12_H_16_N_4_OS, muscone, heptadecanoic acid, spermine, 1-(7-methoxy-2-oxo-2H-chromen-8-yl)-3-methyl-2-oxobutyl acetate, guggulsterone, N-acetylmethionine, orlistat, piperine, esculetin, 16,16-dimethyl prostaglandin A1, kinetin, L-dopa, 11-dehydro thromboxane B2, Dl-3,4-dihydroxymandelic acid, and caffeic acid (Figure 3). These known health-beneficial metabolites exhibited different distribution patterns across different termite castes. Most compounds with antioxidant, anticancer, hypoglycemic, and hypolipidemic properties showed significantly higher expression levels in the reproductive caste compared to the worker caste. In contrast, compounds with anti-inflammatory, antiviral, and antibacterial properties were upregulated in the soldier caste, consistent with previous studies on *Mastotermes darwiniensis* termites [35].

### 2.3. Trophallaxis Fishing Assays

**Figure 4:**
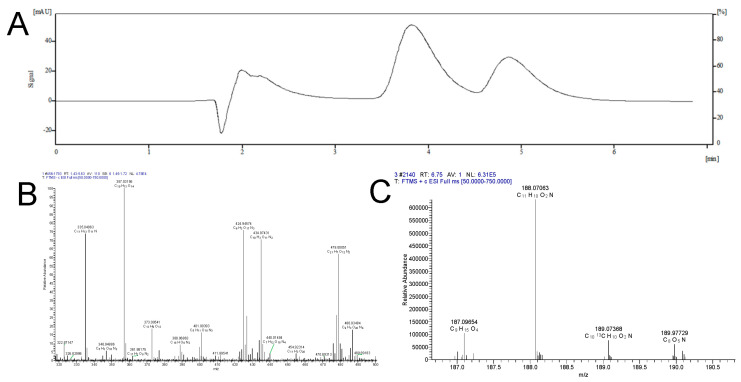
Fish assays. (**A**) MS and MS/MS spectra of the screened compounds. (**B**) Thermo Fisher Scientific database matching results. (**C**) Thermo Fisher Scientific database matching results.

This study selected colonies of *R. flaviceps* that included reproductive castes as research subjects. Through fishing assays and high-throughput analysis, a total of 65 natural compounds were identified (Figure 4). These compounds included adenosine, urocanic acid, uric acid, trans-3-indoleacrylic acid (Figure 4C), bis(4-ethylbenzylidene) sorbitol, metoprolol, methyl palmitate, hexadecanamide, esmolol, choline, N,N′-diphenylguanidine, L-histidine, L-pyroglutamic acid, DEET, minoxidil, DL-arginine, PEG n_11_, C_20_H_26_O_3_, diphenylphosphinic acid, octyl hydrogen phthalate, dodecylamine, C_9_H_19_NO, D-(+)-maltose, C_12_H_27_NO, dibenzylamine, C_16_H_14_O_7_, L-tyrosine, DHEA, NP-019636, PEGn_10_, cytidine, vanillin, methoxyphenamine, uracil, cytosine, C_20_H_30_O_4_, C_12_H_12_N_2_O_2_, palmitic acid, proline, nicotinic acid, L-phenylalanine, NP-020014, C_20_H_28_O_3_, and paroxetine. 13,14-dihydro-15-keto prostaglandin J2, 4-hydroxyphenylpyruvic acid, 3-hydroxy-6-methoxy-2-phenyl-4H-chromen-4-one, diheptyl phthalate, D-(+)-tryptophan, C_11_H_20_O_4_, C_10_H_16_O_5_, phloroglucinol, radicinin, NP-011220, triethyl phosphate, NP-003117, prolinamide, C_15_H_18_O_4_, D-carnitine, D-serine, and C_13_H_22_O_3_. Notably, further comparison with data from metabolomic results revealed that the content of trans-3-indoleacrylic acid (IDA) was significantly elevated in samples from reproductive castes compared to those from worker castes (Figure 3B). This finding highlights the potential importance of IDA and other identified compounds in physiological and behavioral regulation within termite colonies.

### 2.4. Toxicity Test Results

For treatment with 0.05, 0.5, 5, 50 and 500 mg/L IDA solutions, mortality with time is shown in Table 1. When zebrafish were exposed to IDA concentrations of 50 mg/L and higher, such as 500 mg/L, for 24 h, all fish died, resulting in a 100% mortality rate. This clearly indicates that high concentrations of IDA are significantly toxic to zebrafish and unsuitable for further research. Conversely, in lower concentration ranges, zebrafish (15 fish per group, triplicate experiments) were exposed to IDA concentrations of 0.05 mg/L, 0.5 mg/L, and 5 mg/L for 72 h. The mortality rate did not exceed 7%, indicating that these concentrations are relatively safe for zebrafish. It was determined that the safe concentration range for IDA in zebrafish is 0.05 to 5 mg/L (Table 1).

### 2.5. H_2_O_2_ Aging Model Establishment

**Figure 5:**
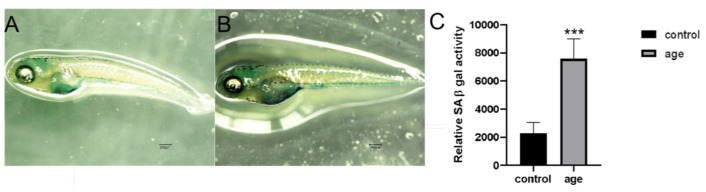
SA-β-gal staining after senescence modeling. (**A**) Control zebrafish SA-β-gal staining results. (**B**) Staining results of the premature aging model. (**C**) Histogram of staining levels of SA-β-gal in control and laboratory groups. Data are means ± SEM; *** *p* < 0.001. Scale bar = 200 μm.

Based on previous experimental methods, we successfully established a prematurely aging zebrafish model using hydrogen peroxide (H_2_O_2_) treatment [36]. To verify the accuracy of this model, we used a senescence-associated β-galactosidase (SA-β-gal) staining kit for detection (Figure 5). The results showed that the tissue samples from the H_2_O_2_-induced premature aging model group exhibited significantly higher SA-β-gal activity compared to the control group (*p* < 0.001).

### 2.6. Expression of Aging-Related Genes

**Figure 6:**
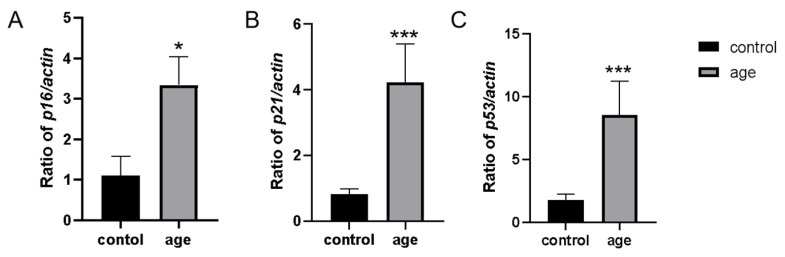
mRNA expression of senescence marker genes *p16*, *p21*, and *p53*. (**A**) Changes in zebrafish *p16* gene expression before and after modeling. (**B**) Changes in zebrafish *p21* gene expression before and after modeling. (**C**) Changes in zebrafish *p53* gene expression before and after modeling. Data are means ± SEM; * *p* < 0.05; *** *p* < 0.001.

Gene expression analysis revealed that in the H_2_O_2_-induced group, the mRNA expression levels of *p16*, *p21*, and *p53* were all significantly increased compared to the control group (*p* < 0.01). This finding is consistent with the elevated SA-β-gal activity observed in the same group, confirming the effectiveness of the H_2_O_2_-induced premature aging model (Figure 6).

### 2.7. Survival Numbers of Prematurely Aged Zebrafish Induced by Different Concentrations of IDA

In this study, we used hydrogen peroxide (H_2_O_2_) to induce a prematurely aging zebrafish model to investigate the effects of IDA on zebrafish lifespan. The experiment monitored the survival status of zebrafish over a 14-day observation period under different concentrations of IDA treatment. By day 12, the survival rate of the control group zebrafish dropped to 0%, indicating that the premature aging conditions induced by H_2_O_2_ are extremely detrimental to zebrafish survival. At 0.05 mg/L IDA, the final survival rate was 15.5%, at 0.5 mg/L IDA, the survival rate increased to 22.2%, and at 5 mg/L IDA, the highest survival rate observed was 28.8%. These data showed that with the IDA concentration increased from 0.05 mg/L to 5 mg/L, the survival rate of the zebrafish improved significantly (Table 2).

### 2.8. mRNA Expression of p21, p16, and p53

**Figure 7:**
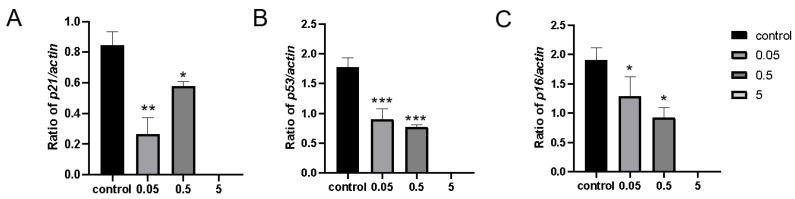
mRNA expression of senescence marker genes *p16*, *p21*, and *p53*. (**A**) Expression changes of the *p21* gene in zebrafish fed with a gradient for 7 days. (**B**) Expression changes of the *p53* gene in zebrafish fed with a gradient for 7 days. (**C**) Expression changes of the *p16* gene in zebrafish fed with a gradient for 7 days. Data are means ± SEM; * *p* < 0.05; ** *p* < 0.01; *** *p* < 0.001.

To investigate whether IDA treatment could extend the lifespan of prematurely aged zebrafish compared to the control group, we conducted a comprehensive qPCR analysis of senescence-related genes. The experiment aimed to assess changes in gene expression after seven days of IDA treatment. The results showed that genes *p21*, *p16*, and *p53* were significantly downregulated after IDA treatment. At higher concentrations of IDA, the down-regulation was more pronounced. Specifically, at 5 mg/L, the expression levels of *p21*, *p16*, and *p53* were reduced to undetectable levels (*p* < 0.001) (Figure 7).

#### 2.8.1. mRNA Expression of *at1* and *at2*

**Figure 8:**
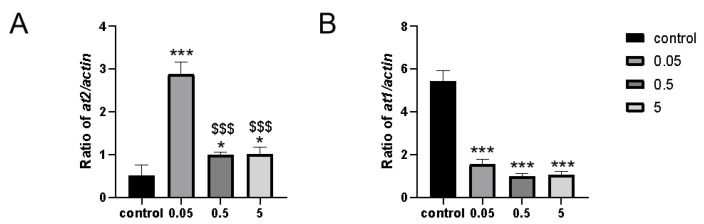
mRNA expression of senescence marker genes *at1* and *at2*. (**A**) Expression changes of the *at2* gene in zebrafish fed with a gradient for 7 days. (**B**) Expression changes of the *at1* gene in zebrafish fed with a gradient for 7 days. Data are means ± SEM. Compared with the control group: * *p* < 0.05; *** *p* < 0.001; compared with the 0.05 mg/L group: $$$ <0.001.

To explore the regulatory pathways by which IDA affects lifespan and to validate the accuracy of our detection method, we examined the expression levels of the key renin–angiotensin system genes *at1* and *at2*. Interestingly, after seven days of IDA treatment, *at2* was significantly upregulated, while *at1* was significantly downregulated [37] (Figure 8). Thus, the result confirms the accuracy of our method and provides evidence that IDA influences the renin–angiotensin system, a pathway known to play a critical role in the aging process.

#### 2.8.2. mRNA Expression of *insulin*, *foxoa*, *igf1* and *mTOR*

**Figure 9:**
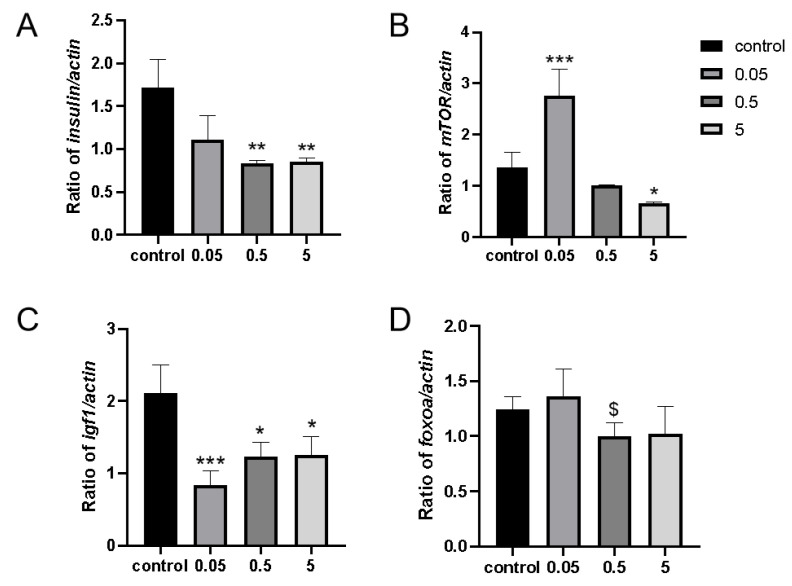
mRNA expression of genes *insulin*, *mTOR*, *igf1*, and *foxoa*. (**A**) Expression changes of *insulin* gene in zebrafish fed with gradient for 7 days. (**B**) Expression changes of *mTOR* gene in zebrafish fed with gradient for 7 days. (**C**) Expression changes of *igf1* gene in zebrafish fed with gradient for 7 days. (**D**) Expression changes of *foxoa* gene in zebrafish fed with gradient for 7 days. Data are means ± SEM. Compare with the control group * *p* < 0.05; ** *p* < 0.01; *** *p* < 0.001. Compared with the 0.05 mg/L group: $ <0.005.

It is well known that changes in nutrient intake can affect lifespan, with the most critical nutrient-sensing pathways being the insulin/IGF-1 signaling (IIS) pathway and the mTOR pathway. To investigate whether IDA modulates lifespan through these two pathways, we examined the expression of key genes involved. After IDA treatment, the expression levels of the key genes *insulin* and *igf1* were significantly reduced, with *igf1* being more sensitive to IDA compared to insulin. However, there was no significant change in *foxoa.* Interesting, mTOR showed opposite results at low and high concentrations. These findings indicate that IDA influences lifespan by regulating the IIS and mTOR pathways, particularly showing significant effects on *igf1* expression and exhibiting concentration-dependent opposite effects on mTOR (Figure 9).

## 3. Discussion

In social insects, trophallaxis was initially thought to primarily function in the exchange of food, such as the transfer of nutrients from foragers to worker ants within the nest and then from these workers to larvae [38]. However, as research has advanced, it has been discovered that trophallaxis also provides outgoing foragers with information about available food sources [39]. In the study of trophallaxis in a social hymenopteran insect, the *Camponotus floridanus* ant, it was found that the regurgitate not only facilitates nutritional exchange and provides information about food sources but also contains specific digestive and non-digestive proteins, hydrocarbons, microRNAs, and key developmental regulators such as juvenile hormone [40]. This research revealed that the trophallactic fluid plays a multifaceted role beyond mere nutrition. Similar findings were observed in a study of termite *R. labralis*, where the trophallactic material also contained a variety of substances in addition to nutritional components. To our knowledge, this is the first study to use metabolomic methods to analyze the trophallactic fluid from all castes except nymphs in an entire termite colony. The detected substances in the trophallactic fluid can be broadly categorized into lipids and lipid-like molecules, organic acids and derivatives, organic heterocyclic compounds, nucleosides, nucleotides and analogues, oxidized compounds, phenylpropanoids and polyketides, alkaloids and derivatives, organic sulfur compounds, nucleic acids, phenolic compounds, and organic nitrogen compounds, as well as small amounts of unknown compounds. Our experimental results indicate that the trophallactic fluid contains not only nutrients but also a wide range of other compounds that likely play roles in the growth and development of the entire colony (Figure 2 and Figure 3). Moreover, the composition of these compounds varies among different castes due to their distinct roles within the colony. For instance, the trophallactic fluid from reproductive castes contains various hormones that influence reproduction, including compounds such as 13,14-dihydro prostaglandin E1, prostaglandin A3, estrone, estrogen, prostaglandin A1, 8-iso-15-keto prostaglandin E2, methyltestosterone, testosterone, and 17α-hydroxyprogesterone (Figure 3).

Recent research on the subterranean *R. speratus* termite has revealed that reproductive castes possess significantly higher levels of coenzyme Q_10_ in both their bodies and intestines compared to worker castes. When CoQ_10_ was supplemented in worker termites under oxidative stress, their survival rates increased [41]. Recent research on *R. speratus* has revealed that reproductive castes possess significantly higher levels of coenzyme Q_10_ in both their bodies and intestines compared to worker castes. When CoQ_10_ was supplemented in worker termites under oxidative stress, their survival rates increased [42]. By comparing our results with the relevant literature and metabolomic data, we identified IDA (Figure 4C). This study shows that IDA helps extend the lifespan of prematurely aged zebrafish, and this effect is concentration-dependent.

In current research fields, indole compounds have become a central focus in studies on gut microbiota and metabolic disorders. Experimental evidence has shown that tryptophan can be metabolized by gut microbiota into indole compounds, which have the ability to improve intestinal epithelial barrier function [43,44]. Further investigation revealed that indoles not only serve as important metabolic products but also function as signaling molecules involved in physiological regulation within the body. Specifically, indoles promote the release of glucagon-like peptide 1 (GLP-1) from intestinal endocrine L cells, thereby indirectly influencing insulin secretion and mechanisms regulating appetite [45]. Additionally, indolepropionic acid, another important metabolite of tryptophan through gut microbiota, is a short-chain fatty acid that contains an indole ring structure. Studies have revealed that it can provide protective effects on β-cells and may reduce the risk of type 2 diabetes (T2D). Moreover, indolepropionic acid exhibits multiple biological activities, including antioxidant, anti-inflammatory, and neuroprotective properties [46]. Notably, IDA exhibits dynamic changes under specific pathological conditions. Research has indicated that acute kidney injury induced by cardiac surgery can lead to increased expression of IDA in plasma [47]. Additionally, other studies have shown that its levels within the gut are correlated with neurodevelopmental processes [48]. Recent studies have further revealed that IDA has the ability to inhibit ferroptosis and may promote the development of colorectal cancer. Ferroptosis is a form of non-apoptotic cell death caused by uncontrolled lipid peroxidation and subsequent membrane damage [49,50]. The preliminary results of this experiment indicate that IDA modulated the lifespan of the experimental subjects through a dose-dependent effect on the IIS (insulin/insulin-like growth factor signaling) pathway and the RAS pathway. Notably, the modulation of the IIS pathway by IDA did not require the involvement of *foxoa* (Figure 8 and Figure 9). Further research is needed to elucidate the mechanisms by which IDA regulates lifespan.

## 4. Materials and Methods

### 4.1. Sample Collection

Termite, *R. labralis* colonies were collected from Shaanxi Province, China (at elevation 575.7 m, longitude 109.1349° E, latitude 34.3350° N; elevation 853.6 m, longitude 109.231204° E, and latitude 34.312488° N) during the period from May to April 2021. Isolated colonies were reared in plastic bottles (5.5 cm × 5.5 cm × 6.5 cm), regularly sprayed with water, and maintained in the laboratory at 25 °C.

### 4.2. Termite Rearing and Induction of Reproductive Caste

Wild colonies of termite *R. labralis* were collected from decaying wood in natural environments. Adult termites (including kings, queens, and supplementary reproductives) and workers were separated. Each group was composed of 100 to 200 workers and 10 to 30 soldiers, and at least 60 such groups were prepared. Under conditions simulating their natural habitat, these groups were maintained at 25 °C in darkness for 30 days to induce workers to transform into supplementary reproductives (kings or queens). The development of the first batch of eggs was observed and recorded until they hatched into larvae and further developed into mature workers. When the first batch of eggs successfully develops into mature workers, this marks the successful establishment of a reproductive caste structure within the colony. At this point, it can be considered that the sampling criteria have been met.

### 4.3. Location of Trophallaxis Fluid

A 1% rhodamine B water solution (Xiang Min Road, Songjiang, China) was used to dye the food source of termite *R. labralis* for 24 h. The dyed food was then dried and set aside for use. Half of the worker termites from a nest colony were selected and fed on the dyed wood chips for 48 h under conditions with ample water and food. After the 48 h feeding period, the original reproductive caste (kings and queens) were introduced into the colony. After an additional 48 h of feeding, the liquid in the filter paper was squeezed out under a microscope to observe whether the liquid in the mouths of reproductive and non-reproductive castes was stained or not. Moreover, following the observation of the staining in the trophallaxis fluids, the termites were dissected to examine which parts of the gut were stained. Each experiment was conducted with three replicates to ensure the reliability of the results.

### 4.4. Trophallaxis Fluid Collection

The female and male termites were examined by the length of their ventral slices, and the age was observed by the number of antenna nodes: male worker (WM), primary reproductive queen (QF), primary reproductive queen (QM), supplementation reproductive queen (NF), supplementation reproductive queen (Nm), female soldier (SM), and male soldier (SF). The samples were placed in a 4 °C environment to paralyze the body. The samples were taken in two ways: fixing the termites with blue gelatin under a microscope and inducing them to spit out part of the oral fluid by stimulation with a self-made, very fine pipette; subsequently, through dissection, the crop and a portion of the digestive tract were extracted. The tissues were then homogenized using liquid nitrogen, followed by centrifugation to obtain the supernatant. The trophallaxis fluids from the oral region were mixed with those from the crop to yield a complete sample. For each group, 30 individuals were collected, resulting in a minimum volume of 10 μL of trophallaxis fluids.

### 4.5. Non-Targeted Metabolomics

The samples to be tested were placed in EP tubes, and 400 μL of an 80% methanol aqueous solution was added for initial dissolution and extraction. Afterwards, the samples were vortexed to ensure thorough mixing. They were then placed on ice for 5 min, followed by centrifugation at 15,000× *g* for 20 min at 4 °C. After centrifugation, the supernatant was aspirated and diluted with mass spectrometry-grade water to achieve a final methanol content of 53%. The diluted solution was again centrifuged under the same conditions (15,000× *g*, 4 °C) for 20 min. The supernatant collected from this step was used as the sample for subsequent LC–MS analysis. The processed samples were analyzed using an LC–MS instrument from Thermo Fisher (Karlsruhe, Germany). The scan range was set between *m*/*z* 100 and 1500. The electrospray ionization (ESI) source parameters were as follows: spray voltage set to 3.5 kV, sheath gas flow rate at 35 psi, auxiliary gas flow rate at 10 L/min, ion transfer tube temperature set to 320 °C, ion injection RF level at 60, auxiliary gas heater temperature at 350 °C, and the instrument was operated in either positive or negative polarity mode for analysis. The secondary scans were conducted in a data-dependent acquisition mode.

### 4.6. Synthesis of SiO_2_@Ag

The synthesis of SiO_2_@Ag nanocomposites was based on the Stöber method [51]. First, silica nanoparticles were prepared according to the Stöber method, i.e., 1.2 mL of ethyl orthosilicate (TEOS), 16 mL of anhydrous ethanol, 6 mL of ammonia, and 25 mL of deionized water were mixed and reacted under magnetic stirring conditions to generate silica cores. Next, in order to obtain silver-modified SiO_2_@Ag core–shell-structured nanoparticles, the silica nanoparticles obtained from the above synthesis were first dispersed in 50 mL of distilled water using ultrasonic waves to form a homogeneous and stable suspension. Then, 7.6 mg of silver nitrate as a silver source, 9.1 mL of polyvinylpyrrolidone as a stabilizer and reducing agent, and 5.1 mg of sodium citrate as a complexing agent for silver ions were added to this suspension to control the growth and morphology of silver nanoparticles. Finally, the mixture was placed in a thermostat with continuous stirring at 70 °C for one hour to reduce and uniformly deposit silver ions on the silica surface, which successfully formed a silver shell layer on the surfaces of the silica nanoparticles and ultimately yielded the desired SiO_2_@Ag core–shell structured nanoparticles.

### 4.7. Expression of AT2 Protein in Escherichia coli

In this experiment, a βAR (β-adrenergic receptor) plasmid with a Halo tag at the C-terminus was constructed in *E. coli* host cells by fusing the BP (recombinase-mediated sticky end joining) and LR (homologous recombination) reaction techniques in the bioorthogonal reaction. This plasmid expression clone was further transferred into the *E. coli* BL21 (DE3) strain, and positive clones were screened by ampicillin resistance. Colonies were picked from individual clones and cultured in LB liquid medium at 37 °C overnight and then transferred to self-inducing medium containing inducing agents to continue culturing for 12 h to induce the target protein expression. Finally, the bacterial precipitate was collected by centrifugation, suspended, and incubated at 37 °C for 30 min to fully lyse the cells, after which the cells were broken down by ultrasonic crushing in an ice bath and then centrifuged to separate the supernatant and precipitate for subsequent extraction and purification of the recombinantly expressed Halo-tagged β2-AR protein.

### 4.8. Fixed AT2 Protein

In this experiment, AT2 molecules were successfully immobilized on SiO_2_@Ag core–shell nanostructures by employing a bioorthogonal torrefaction reaction. The obtained immobilized probes were dissolved in 1.0 mL (20 mM, pH = 7.4) of phosphate buffer and stored at 4 °C to ensure their stability and subsequent application performance.

### 4.9. Screening of Trophallaxis Fluid for Compounds That Interact with Immobilized AT2 Proteins

A liquid chromatography–mass spectrometry (LC–MS) column (Agilent, SL1100, Shanghai, China) equipped with a binary pump and a column temperature chamber was used in this study, and ChemStation software version 5.2 was used for data processing and acquisition. The column used was an immobilized β2-adrenoceptor (β2-AR)-specific column with the following specifications: particle size of 7.0 μm, pore size of 300 Å, and size of 30 × 4.6 mm. In the liquid chromatography section, the mobile phase was selected as a 20 mmol/L ammonium acetate buffer solution, and the pH value was adjusted to 7.2 in order to optimize the interaction between the target compounds and the stationary phase and to achieve the effective separation. For the mass spectrometry section, the specific conditions were set as follows:

The ion spray voltage was set to −4500 volts to facilitate the ionization process. The pressure of the atomizer gas (nitrogen, N_2_) was adjusted to 35.0 pounds per square inch (psi) to ensure that the sample was sufficiently atomized to form ions, the flow rate of the drying gas (also nitrogen, N_2_) was set to 8.0 L/min (l min^−1^) to remove solvent molecules from the sample ions, and the drying gas temperature was set to 350 °C to ensure rapid evaporation of solvent and avoid unnecessary ion fragmentation. In order to quantify the components in the cross-feeds, the selected ion-pair method was used, and the injection volume was set to 5.0 μL. During the experiments, the liquids corresponding to the peaks collected sequentially were transferred to a freeze dryer for drying and processing, so as to facilitate the further identification and quantitative analysis of the substances.

### 4.10. High-Performance Liquid Chromatography (HPLC)

The screened cross-feeder lyophilized powder was allowed to stand at 4 °C for 30 min to stabilize its structure and reduce the effect of temperature change on the sample. A 200 mg sample of lyophilized powder was weighed and added to 100 μL of liquid-grade purified water precooled to a low temperature (usually 4 °C) for initial dissolution. Afterwards, 400 μL of precooled methanol acetonitrile solution was further added to the above solution to enhance the solubility and extraction efficiency of the target compounds. The samples were brought into full contact with the solvent by blowing and mixing and placed in an ultrasonic shaker for two ultrasonic treatments of 30 min each in order to break up the cells or particles more thoroughly and to promote the release of the target substances and homogeneous mixing. The obtained sample solution was allowed to stand at −20 °C for 1 h to induce the precipitation of certain components insoluble in organic solvents. The supernatant and precipitate were then separated by centrifugation at 12,000 r/min for 20 min at 4 °C. The supernatant was dried in a freeze dryer to remove the solvent, and the concentrated target compound was obtained. The dried product was redissolved in 200 μL of acetonitrile and centrifuged at 12,000 r/min for 15 min at 4 °C to ensure that the solution was clear and free of particles, and then the supernatant was collected again for subsequent detection. The supernatant was put into a liquid chromatograph for detection. Analytical conditions: The column was a Hypersil Gold 100 × 2.1 mm liquid chromatography column with a particle size of 3 μm, manufactured by Thermo Fisher Scientific (Waltham, MA, USA) and operated at 25 °C. The mobile phases: mobile phase A was methanol, and mobile phase B was a 0.1% formic acid aqueous solution. A gradient elution strategy was used: the time gradient was set from 0 min to 4 min, 12 min, 14 min, 14.1 min and 20 min, and the percentage of mobile phase A (% A) was 5%, 5%, 70%, 70%, 5%, and finally recovered to 5%, respectively. The flow rate was maintained at 0.25 mL/min during gradient dehydration. The injection volume for each injection of 2 μL of the sample solution to be tested was injected into the liquid chromatograph for analysis.

#### Mass Spectrometry (MS)

Instrument: Thermo Fisher Scientific Q Exactive Mltimate 3000 UPLC; conditions: sheath gas 40.00 Arb; auxiliary gas: 8.00 Arb; spray voltage: 3200 v; capillary temperature: 300.00; max. spray current: 100.00 µA; probe heater temperature: 300.00 °C; ion source: esi ± ms; wavelength: 190–400 nm; flow rate = 0.25 mL/min.

### 4.11. Zebrafish Breeding

Wild-type zebrafish, *Danio rerio* were purchased from the Guangdong aquatic pet market. To ensure that the zebrafish were in the best physiological condition during the experiment, all zebrafish were kept in a circulating water system at a constant temperature of 28 °C for more than two weeks before the experiment, with a natural light/dark cycle of 14 h of light and 10 h of darkness per day. The day before the experiment, healthy, active, sexually mature zebrafish with a sex ratio of 1:2 (females to males) were selected and placed in a special zebrafish mating box. On the day of the experiment, the researchers turned on the light stimulus to induce the zebrafish to enter a reproductive state, allowing the zebrafish to mate and spawn freely. After one to two hours, the zebrafish would form transparent oval-shaped eggs on the bottom of the water. At this point, the experimenter collects these freshly laid zebrafish eggs using a rubber-tipped dropper for subsequent experiments.

### 4.12. Toxicity Testing

Healthy zebrafish embryos were collected, and when the zebrafish eggs successfully hatched, they were placed in IDA solutions containing different concentration gradients of 0.05 mg/L, 0.5 mg/L, 5 mg/L, 50 mg/L, and 500 mg/L until the end of the toxicity experiment.

### 4.13. Significant Aging Agent Treatment

Healthy zebrafish embryos were selected during a window of 4 to 6 h after hatching, which is an important period of zebrafish development. These embryos were briefly exposed to a solution containing 2 mmol/L hydrogen peroxide (H_2_O_2_) for 6 h. At the end of the exposure, treated zebrafish samples were rapidly collected for subsequent molecular biology assays.

### 4.14. SA-β-Gal Test

Zebrafish incubated normally for 72 h and prematurely aged zebrafish induced with senescence inducers were removed, and both groups were placed in cold water at 4 °C using a cryogenic method to cause rapid death, followed by rapid fixation of the samples with 4% paraformaldehyde fixative. The fixation time was controlled to be within 10 to 15 min. After fixation, the samples were rinsed three times with phosphate buffer solution (PBS) for 5 min each time, and the cleaned samples were immersed in SA-β-gal staining solution and incubated overnight at 37 °C to detect senescence-associated β-galactosidase activity. At the end of incubation, the staining solution was discarded, and the samples were washed again with PBS twice, each time for 5 min. Finally, the samples were observed and photographed by microscope.

### 4.15. Total RNA Extraction and Real-Time PCR

Total RNA was extracted using Trizol (Thermo Fisher Scientific, Waltham, MA, USA). After obtaining RNA, absorbance was measured at 260 and 280 nm to determine concentration and purity. Then, reverse transcription was performed using 1 μg of total RNA. RNA concentration was determined in time for the reverse-transcription assay. Reverse transcription of RNA was performed in two steps, i.e., genomic DNA removal and reverse transcription, using a reverse-transcription kit (Accurate Biology, Changsha, Hunan, China). After the reaction, the samples were stored at −20 °C. All the procedures were manipulated in accordance with the manufacturer’s protocol. The sequences of the PCR primers used are shown in Table 3.

### 4.16. Lifespan Test

Zebrafish embryos were exposed to 2 mmol/L H_2_O_2_ for 6 h after 72 h of fertilization to construct a senescence model. After that, zebrafish were divided into four groups: one control group (no IDA was added to the control group) and three experimental groups (exposed to IDA according to the results of the toxicity experiment in a gradient), with 15 fish in each group. The three experimental groups had IDA added in a gradient. The IDA gradient solutions were 0.05 mg/L, 0.5 mg/L, and 5 mg/L. The number of days of survival for each group was recorded.

### 4.17. Statistical Analysis

The identified metabolites were annotated using the KEGG database, HMDB database, and Lipid Maps database. In the multivariate statistical analysis section, the data were transformed using metaX metabolomic data processing software and subjected to principal component analysis (PCA) and partial least squares discriminant analysis (PLS-DA) to obtain VIP values for each metabolite. For the univariate analysis part, statistical significance (*p* value) of each metabolite between the two groups was calculated based on the *t*-test, and the fold change of metabolites between two groups was calculated, i.e., the FC value. The default criteria for differential metabolite screening were VIP > 1, *p* < 0.05, and FC ≥ 2 or FC ≤ 0.5.

The raw LC-MS data were first processed and analyzed using the metabolomic software Progenesis QI v2.0 (provided by Waters Corporation, Milford, MA, USA). Specific steps included baseline correction to reduce the influence of background noise, automatic peak identification and accurate quantification, retention time calibration to achieve peak alignment between different samples, and other pre-processing operations. After this series of rigorous processing, a detailed data matrix containing retention time, mass-to-charge ratio (*m*/*z*), and peak intensity for each compound was successfully generated. In order to further clarify the identity of the obtained compounds, the information in the above data matrix was comprehensively and accurately compared with two internationally recognized public metabolite databases, http://humdb.ca/metabolites and https://metlin.scripps.edu/. PCR statistical analyses were performed using IBM SPSS Statistics version 22.0, and graphs were drawn using GraphPad Prism 8.0. The normal distribution of the data was tested using Fisher’s exact method. For weights, the data were analyzed using *t*-tests for dependent significant differences. Differences were considered significant when the *p*-value was * *p* < 0.05.

## 5. Conclusions

This is the first study to use nanogram-level liquid chromatography–mass spectrometry (LC–MS) technology to conduct a comprehensive and in-depth metabolomic analysis of the trophallactic fluid from different caste members within a colony of the termite *R. labralis*. We successfully identified 672 metabolites in positive ion mode and 356 metabolites in negative ion mode. Based on this, leveraging existing KEGG (Kyoto Encyclopedia of Genes and Genomes) database resources, we screened out seven known natural compounds closely related to aging regulation pathways. Using a “fishing assay” approach, which involves the synthesis of SiO_2_@Ag nanoclusters coated with 6-chlorohexanoic acid for enzyme substrate recognition, we successfully isolated a series of natural compounds that interact with the AT2 receptor. By comparing these screening results with previous experimental data and referencing relevant scientific literature, we preliminarily confirmed that trans-3-indoleacrylic acid may be a newly discovered key compound closely associated with lifespan regulation in this experiment. The validation was conducted using a prematurely aged zebrafish model. Preliminary results indicate that IDA modulated the lifespan of the experimental subjects through a dose-dependent effect on the IIS (insulin/insulin-like growth factor signaling) pathway and the RAS pathway. Notably, the modulation of the IIS pathway by IDA does not require the involvement of *foxoa*.

## Figures and Tables

**Table 1:** Toxicity test results.

Death (F) Group Day	0.05 mg/L	0.5 mg/L	5 mg/L	50 mg/L	500 mg/L
1	0	0	1	15	15
1	0	0	1	15	15
1	0	0	1	15	15
2	0	0	0	0	0
2	0	0	0	0	0
2	0	1	0	0	0
3	0	0	0	0	0
3	0	0	0	0	0
3	1	0	0	0	0

**Table 2:** Lifetime experiment survival rate.

Time	0 mg/L (%)	0.05 mg/L (%)	0.5 mg/L (%)	5 mg/L (%)
1	100	97.7	97.7	100
2	97.7	97.7	97.7	100
3	97.7	95.5	97.7	97.7
4	97.7	95.5	97.7	97.7
5	77.7	80.0	91.1	91.1
6	64.4	66.6	68.8	68.8
7	40.0	35.5	48.8	51.1
8	31.1	22.2	33.3	31.1
9	28.8	15.5	24.4	28.8
10	22.2	15.5	24.4	28.8
11	13.3	15.5	22.2	28.8
12	0	15.5	22.2	28.8
13	0	15.5	22.2	28.8
14	0	15.5%	22.2	28.8

**Table 3:** Primer information.

Gene	Forward Primer	Reverse Primer
*actin*	5’-CCACCATGTACCCTGGCATT-3’	5’-CATCTGCTGGAAGGTGGACA-3’
*p21*	5’-CTGCACTCCCGCATGAAG-3’	5’-GACGCTTCTTGGCTTGGTAGAA-3’
*p16*	5’-AACGTCGAGGATGAACTGACC-3’	5’-CAAGAGCCAAAGGTGCGTTA-3’
*p53*	5’-TAAGAGTGGAGGGCAATCAGC-3’	5’-GCACAGTTGTCCATTCAGCA-3’
*mTOR*	5’-TTACGACAGGACGAGAGGGT-3’	5’-GAGTTGGTGGAAAGCGGGAT-3’
*igf1*	5’- GTACCCACACCCTCTCACTG-3’	5’-GTCCATATCCTGTCGGTTTGC-3’
*insulin*	5’-GGCCCAACAGGCTTCTTCTA-3’	5’-ATGCAAAGTCAGCCACCTCA-3’
*atg2*	5′-CGACCTGCTACAGCCGAATC-3′	5′-TGCAACACATGAACCAACCG-3′
*atg1*	5′-TGCGTTCTAGTTTGGGTGGT-3′	5′-GACTGAGAGCTGCAAGGACA-3′
*foxoa*	5’-GTTTGCCAAGAGCAGAGGAC-3’	5’-CATTGCTGTGGGAGTTCGGA-3’

## Data Availability

Experimental data are available from the corresponding author on request.

## Supplementary Materials

The supporting information can be downloaded at: https://www.mdpi.com/article/10.3390/ijms26041543/s1.
